# Effect of excessive gestational weight on daughters’ breast density at the end of puberty onset

**DOI:** 10.1038/s41598-020-63260-9

**Published:** 2020-04-20

**Authors:** Ana López, María Luisa Garmendia, John Shepherd, Karin Michels, Camila Corvalán, Ana Pereira

**Affiliations:** 1 Master in Nutrition Program, Institute of Nutrition and Food Technology, University of Chile, Santiago, Chile; 2 Institute of Nutrition and Food Technology, University of Chile, Santiago, Chile; 3 Population Sciences in the Pacific Program, University of Hawaii Cancer Center, Honolulu, Hawaii USA; 4 Department of Epidemiology, Fielding School of Public Health, University of California, Los Angeles, California USA; 5 Institute for Prevention and Cancer Epidemiology, Faculty of Medicine and Medical Center, University of Freiburg, Freiburg, Germany

**Keywords:** Cancer, Medical research

## Abstract

The effect of excessive gestational weight gain (EGWG) is related to adverse health outcomes in the offspring; however, its effect on the daughters’ breast density is unclear. We aimed to assess the association between EGWG and daughters’ breast composition (% of fibroglandular volume (%FGV) and absolute fibroglandular volume (AFGV)) at Tanner stage 4 (Tanner B4)). We included 341 girls and their mothers from an ongoing cohort of low-income Chilean girls born from 2002–2003. Maternal gestational weight gain was self-reported in 2007, and breast density by digital mammography was measured in 2010. Weight, height and breast composition by dual X-ray absorptiometry (DXA) were measured in daughters at Tanner B4. Logistic regression models were run to assess the association between EGWG and the 80th percentile of %FGV and AFGV. Mean gestational weight gain was 13.7 kg (SD = 6.9 kg). Women with pregestational overweight or obesity exceeded the recommended gestational weight gain (58.8% vs. 31.8%, respectively). Daughters of women who had EGWG had higher levels of AFGV (OR: 2.02; 95%CI 1.16–3.53) at Tanner B4, which could be explained by metabolic and hormonal exposure in utero. However, we did not observe an association with %FGV.

## Introduction

High mammographic breast density (BD) is one of the most important determinants of breast cancer (BC) risk; women with dense breasts (≥75%) have a 4.6-fold (3.6–5.9) higher risk of BC than women with non-dense breasts (<5%)^1^. The dense tissue corresponds to epithelial and connective tissue, which is vulnerable to the effect of carcinogenetic agents and potential mutations. Studies in young populations suggest that the percent of BD is highest at young ages (end of puberty) and declines thereafter^2^. The total amount of dense tissue increases during puberty until the breast reaches Tanner stage 4 (B4). At B4, the %BD is highest and subsequently decreases in breast Tanner stage 5 (B5) due to fat infiltration in the breast^3^.

Chile has a high prevalence of overweight and obesity during pregnancy (60.9%)^4^; the mean gestational weight gain (GWG) is 13.7 kg (SD = 7.9 kg), and 43.6% of pregnant women experience excessive gestational weight gain (EGWG)^5^ as defined by the 2009 Institute of Medicine (IOM) guidelines^6^. EGWG is associated with high birth weight (BW) and long-term consequences for the newborn, such as obesity, type 2 diabetes mellitus and cardiovascular disease^7–12^. However, the relation between maternal EGWG and BD in daughters has been little explored. The Early Determinants of Mammographic Density (EDMD) study conducted in 2016 did not demonstrate an association between maternal GWG and BD in daughters (mean age at BD exam = 43 years)^13^. Limited evidence has suggested that EGWG increases BC risk in the offspring; only one study published in 1998 observed that daughters of mothers who gained 11 to 15 kg during their pregnancy had a 50% higher risk of BC than daughters of mothers who gained less than 11 kg^14^, while other study did not find an association^15^. Additionally, epidemiologic studies suggested that for each 1 SD or 1 kg increase in BW, the risk of pre- and postmenopausal BC increases by 6 and 7%, respectively^16,17^. BW has also been associated with BD in women; a 1 SD increase in BW (473 g) resulted in a 3% increase in dense tissue at age 21 y^18^.

Understanding early determinants of breast composition during puberty could contribute to identifying risk factors that may modulate a higher BD at early ages and decrease BC risk in adulthood. To date, no study has evaluated the relation between EGWG and BD at puberty in the offspring. One of the major difficulties is the challenge of measuring BD in young women. Recently, dual energy X-ray absorptiometry (DXA) technology has been developed for this purpose and has been shown to be valid, reproducible and accurate (precision of 2.8%)^19^ with a minimal dose of radiation; therefore, DXA can be used in young women^20^. Thus, by investigating a Chilean cohort of mothers and daughters, our study aimed to assess the relation between EGWG according to the 2009 IOM guidelines^6^ and BD estimated by DXA in the offspring at the end of puberty (B4).

## Results

The mean age of the daughters was 11.2 y (SD: 0.9 y) at the Tanner B4 visit, and 25.8% had experienced menarche at the time of the DXA visit. The mean weight of the daughters was 44.7 kg (SD: 8.7 kg), 49.8% had excessive weight (BMI Z-score> 1 SD), the mean waist circumference was 71.5 cm (SD: 8.9 cm), and a 15.5% had central obesity. In the mothers, the mean age at pregnancy was 27.1 years (SD: 7.6), 34.2% were overweight or obese at the start of their pregnancy, the mean GWG of the mothers was 13.7 kg (SD: 6.9 kg), and 41.9% of the mothers gained more weight than recommended by the IOM 2009 guidelines (Table 1).

**Table 1:** Characteristics of 341 girl and mother participants in this study.

**Girls**	
**At Birth**	
Birth weight (Kg) [mean, SD]	3.3 (0.4)
**At B4**	
Age (years) [mean, SD]	11.2 (0.9)
Already menarche [n, (%)]	88 (25.8)
Weight (kg) [mean, SD]	44.7 (8.7)
Height (cm) [mean, SD]	148 (0.1)
Body mass index (kg/m^2^), [mean, SD]	20.2 (3.3)
Body mass index Z score [mean, SD]	0.9 (1.1)
Nutritional Status (n, %)	
Underweight [<−1 SD]	17 (5.1)
Normal [-1; 1 SD]	151 (45.1)
Overweight [1; 2 SD]	118 (35.2)
Obese [2; 3 SD]	47 (14.0)
Severe obese [>3 SD]	2 (0.6)
Waist circumference (cm) [mean, SD]	71.5 (8.9)
Central Obesity (>90 percentile) [n, (%)]	53 (15.5)
**Mothers at pregnancy**	
Mothers age at the beginning of pregnancy	27.1 (7.6)
Education [n, (%)]	
<12 years	107 (30.9)
12 years	142 (41.0)
>12 years	97 (28.0)
Parity [n, (%)]	
1 living birth	142 (41.4)
+1 living birth	199 (58.4)
Pre-gestational nutritional status (kg/m^2^), [n, (%)]	
Underweight (<18.5)	17 (5.0)
Normal (18.5–24.9)	207 (60.7)
Overweight (25.0–29.9)	93 (27.2)
Obese (≥30.0)	24 (7.0)
Gestational weight gain (kg) [mean, SD]	13.7 (6.9)
Gestational weight gain (kg) by pre gestational nutritional status [mean, SD]	
Underweight (<18.5)	15.6 (10.2)
Normal (18.5–24.9)	13.8 (6.5)
Overweight (25.0–29.9)	13.4 (7.3)
Obese (≥30.0)	12.1 (6.1)
Gestational Diabetes Mellitus [n, (%)]	16 (4.7)

**Figure 1:**
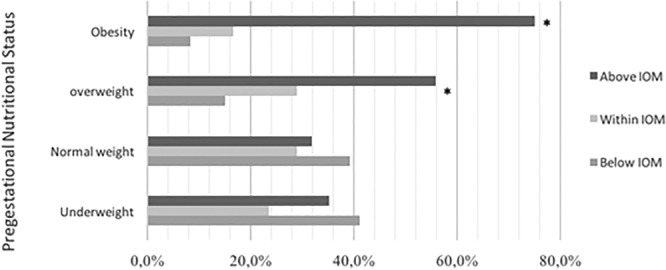
Adherence to IOM gestational weight gain recommendations in relation to pre-gestational nutritional status. *Statistically significant results (Fisher’s test p < 0.05) for excessive gestational weight gain in women with pre-pregnancy BMI of overweight and obesity versus normal pre-pregnancy BMI.

A total of 55.9% and 75% of women who were overweight or obese at the start of their pregnancy, respectively, exceeded the weight gain recommendation. However, only 31.9% of the women who started their pregnancy with a normal pre-gestational BMI exceeded the recommendation; the differences between the groups in both cases were statistically significant (p < 0.05) (Fig. 1).

In the total sample, the median %FGV was 39.0% (IQR = 27.5–54.2) and the median AFGV was 79.5 cm^3^ (IQR = 60.2–100.0). The girls who had menarche prior to the Tanner B4 visit had a significantly higher AFVG than their counterparts. The daughters of the mothers who were underweight prior to their pregnancy had a significantly lower AFGV than the daughters of the mothers with normal pre-gestational nutritional status; in contrast, the daughters of the mothers who were obese prior to their pregnancy exhibited a lower %FGV than the daughters of the mothers in the normal pre-gestational weight category. The daughters of the mothers with GWG exceeding the IOM guideline recommendations (independent of pre-gestational nutritional status) had non-significantly higher AFGV during puberty and a lower %FGV. Additionally, we did not observe conclusive results after stratifying by pre-gestational nutritional status, but the daughters of the women who began their pregnancy in the normal or overweight category and exceeded the recommended weight gain had non-significantly higher AFGV than the daughters of the women who met the recommendations (Table 2).

**Table 2:** Median and Interquartile range (IQR) for breast density measures of girls in relation to study variables.

Variable	N	%FGV	AFGV (cm^3^)
**Total**	341	39.0 (27.5–54.2)	79.5 (60.2–100.0)
**Menarche**			
Yes	88	40.2 (31.6–55.4)	92.6 (75.0–114.2)*
No	253	38.0 (25.9–54.1)	72.00 (57.7–94.3)
**Maternal pre gestational nutritional status**			
Underweight (≤18.5)	17	45.1 (37.1–52.9)	63.4 (52.6–72.0)**£**
Normal weight (18.5–24.9)	204	40.4 (29.1–56.1)	80.4 (59.5–99.7)
Overweight (25.0–29.9)	93	36.6 (26.8–52.1)	80.8 (62.4–105.0)
Obese (≥30.0)	24	26.4 (22.1–33.9)**£**	72.8 (63.8–95.6)
**GWG according to IOM 2009 guidelines during pregnancy**			
Above	141	35.9 (26.5–51.3)	84.4 (64.2–108.9)
Within	94	39.7 (28.8–56.9)	77.1 (60.0–97.4)
Below	102	41.5 (30.1–52.9)	74.2 (57.2–95.2)
**Adherence to IOM recommendation by pre gestational nutritional status**			
Underweight			
- Above	6	41.1 (29.3- 48.7)	61.1 (49.2-72.0)
- Within	5	40.1 (31.7–47.6)	66.2 (63.5–82.8)
- Below	6	46.1 (44.2–52.9)	56.1 (41.8–63.0)
Normal weight			
- Above	65	40.3 (28.8- 57.0)	86.7 (65.2 - 108.9)
- Within	59	38.8 (28.8–57.5)	81.0 (59.1–100.5)
- Below	80	41.8 (30.6–54.3)	75.0 (57.4–95.8)
Overweight			
- Above	52	36.0 (26.3- 50.8)	85.0 (67.6 -110.6)
- Within	27	43.7 (30.3–57.0)	74.4 (62.3–102.6)
- Below	14	33.1 (25.3–49.4)	74.4 (62.3–95.2)
Obesity			
- Above	18	26.3 (22.0 -35.5)	74.0 (64.2 -95.9)
- Within	4	26.8 (22.0–28.1)	68.7 (60.0–80.8)
- Below	4	36.2 (23.9–48.5)	88.0 (71.1–104.9)
**Gestational diabetes**			
- Yes	16	39.6 (22.8 -67.6)	89.8 (68.9-116.2)
- No	322	38.9 (27.4 -53.8)	79.2 (59.6 -100.0)

*Mann Whitney test: significant differences are shown between groups (p < 0.05).

^£^Kruskall Wallis test: normal weight category was used to compare the level of significance between the groups (p < 0.05).

We observed a positive linear relation between GWG in kg and AFGV in the crude and adjusted models (including pregestational weight gain, girl’s BMI z score at time of DXA and maternal education, breast density, parity and gestational diabetes), and even after adjusting for birth weight. The daughters of the mothers who had GWG greater than the recommendations had a 2.3-fold greater risk of having an AFGV over the 80th percentile in the adjusted models (adjusted OR: 2.27, CI 95%: 1.17–4.40) compared to the daughters of the mothers who had a GWG below the recommendations (Table 3). Similar results were observed after further adjusting for birth weight. When comparing the daughters of mothers with EGWG vs. those of mothers with non-EGWG, we observed that the daughters of the mothers who had EGWG had a 2-fold higher risk of an AFGV over the 80th percentile in the crude and adjusted models (adjusted OR: 2.10, 95% CI: 1.19–3.72) (Table 3). We did not observe conclusive results for %FGV (Table 3), as well, we did not observe an association between gestational weight gain and total breast volume (data not shown in tables). BW was inversely correlated with %FGV only in the crude analysis; we did not observe an association with AFGV (Table 3). Additionally, we repeated the analysis using AFGV and %FGV as continuous variable. Similar results and significant results were observed between GWG in kilograms and AFGV and borderline with EGWG and AFGV; no associations with %FGV (supplementary table S1).

**Table 3:** Association of Gestational Weight Gain (in kg and stratified by IOM), birth weight and breast composition (%FGV, AFGV): Crude and adjusted OR and 95%CI.

	FGV% 80^th^ (OR, 95%CI)				AFGV 80^th^ (OR, 95%CI)			
	Crude model	Adjusted model 1	Adjusted model 2	Adjusted model 3	Crude model	Adjusted model 1	Adjusted model 2	Adjusted model 3
Gestational weight gain in kg. **	0.97 (0.94–1.01)	0.99 (0.95–1.03)	0.98 (0.93–1.03)	0.98 (0.93–1.03)	1.04 (1.01–1.07)*	1.05 (1.01–1.08)*	1.06 (1.02–1.10)*	1.06 (1.02–1.11)*
**Gestational weight gain according to IOM**								
Below	1.00	1.00	1.00	1.00	1.00	1.00	1.00	1.00
Within	0.85 (0.49–1.50)	0.76 (0.36–1.60)	0.67 (0.28–1.60)	0.68 (0.28–1.66)	1.02 (0.57–1.83)	1.17 (0.63–2.15)	1.26 (0.62–2.58)	1.38 (0.66–2.86)
Above	0.58 (0.34–0.98)*	0.75 (0.38–1.48)	0.68 (0.30–1.53)	0.69 (0.30–1.66)	1.76 (1.04–2.97)*	1.84 (1.06–3.20)*	2.27 (1.17–4.40)*	2.47 (1.24–4.92)*
**EGWG vs not EGWG**	0.63 (0.40–0.98)*	0.85 (0.47–1.53)	0.82 (0.40–1.65)	0.82 (0.40–1.70)	1.74 (1.12–2.7)*	1.70 (1.07–2.71)*	2.02 (1.16–3.53)*	2.10 (1.19–3.72)*
**Birth weight (in kg.)**	0.52 (0.30–0.91)*	1.07 (0.51–2.25)	1.15 (0.47–2.80)	**—**	1.06 (0.61–1.83)	1.10 (0.61–2.00)	0.89 (0.44–1.80)	**—**

*Model was statistically significant (p value < 0.05).

Adjusted model 1 Adjusted by maternal variables: education, parity, gestational diabetes mellitus; & Girls variables at time B4: BMI Z score and presence of menarche).

Adjusted model 2: Model 1 + maternal breast density.

Adjusted model 3: Model 2 + birth weight (in kg.).

**Gestational weight gain models were adjusted by pre gestational nutritional status.

## Discussion

In our study, we found a positive association of absolute GWG and GWG above the recommended range of the IOM 2009 guidelines with the daughters’ total AFGV, after adjusting for the girls’ BMI, menarche, maternal BD, maternal education, parity, and gestational diabetes mellitus. The daughters of the mothers who had GWG above the recommendation had a twofold-increased risk of having AFGV above the 80^th^ percentile. We did not observe any association between GWG and %FGV of the daughters.

To date, only one study has analyzed the effect of GWG on the BD of daughters^13^. The EDMD study did not observe an association of pre-pregnancy BMI or maternal GWG with %BD or absolute dense breast tissue in the daughters. Michels *et al*. only revealed an increase in %BD in the daughters of mothers who gained ≤5 kg during pregnancy compared with the daughters of the mothers who gained 5 to 10 kg (β = 4.8, 95% CI: 1.0 to 8.6). These authors measured BD in adult women (mean age 43 years (SD = 2.3)); thus, BD may have been modified by known determinants of BD during the life course such as parity, age, breastfeeding, among others, while our study measured BD at Tanner B4, the time with the highest %FGV according to the literature^3^. Additionally, in our study, we observed a higher prevalence of excessive pre-gestational nutritional status and overall maternal GWG [34.2% and 13.7 kg (SD: 6.9 kg), respectively] compared to the findings of the study conducted by Michels *et al*. that reported values of 25% and 9.2 kg (SD = 4.0), respectively. However contemporary follow-up studies are needed to evaluate the association between EGWG and daughter’s breast cancer risk.

Two previous studies have reviewed the association between maternal GWG and the risk of BC in the offspring with divergent results. In 1998, Sanderson *et al*. showed a 50% increased risk of BC in women under 45 years whose mothers gained between 11 and 15 kg during pregnancy (adjusted OR = 1.5, 95% CI 1.1; 2.0) compared with the women whose mothers gained 6.8–10.8 kg; however, they did not observe an association between GWG greater than 15 kg and BC^14^. In 2011, using the data from NHS I and II (n = 2621), Wilson and colleagues did not observe an association between maternal GWG and pre- and postmenopausal BC in the daughters^15^. However, both studies included women with a lower mean GWG compared to those included in our report. In both studies, as well as ours, maternal GWG was determined by self-report, which could have been affected by recall bias, but we expect to be a non-differential measurement error. The time between pregnancy and obtaining data on maternal GWG was shorter in the GOCs cohort (<10 years), reducing measurement error.

Birth weight is a recognized risk factor for BC^16,17^, and women with higher GWG are more likely to have heavier offspring^7–12,21^. Thus, a potential pathway between GWG and BD may be mediated through BW. An EGWG will determine a hormonal environment that makes the offspring prone to higher BW, which is characterized by chronic hyperglycemia, hyperinsulinemia, and insulin resistance^22–25^, as well as higher levels of IGF-1 and leptin^26–29^. Additionally, EGWG and higher pre-gestational BMI are associated with a decrease in SBHG and an increase estrogen bioavailability^30^. However, we did not observe an association between BW and AFGV and only observed an association of BW with %FGV in the unadjusted model. The relation between GWG and AFGV was not modified by including BW as an intermediate of the association (Table 3); thus, suggesting that BW was not a mediator in this association. Additionally, we did not observe an association between gestational diabetes and %FGV or AFGV. However, the lack of association could be due to recruitment restrictions, for example, BWs <2500 or >4000 g were excluded, thus limiting the range to detect an association and the data regarding gestational diabetes was self-reported, thus the possibility of residual confounding remained. Additionally, we did not have data of insulin levels during pregnancy. The positive association we identified between GWG and AFGV may increase BC risk in the offspring; according to Trichopoulos, the presence of IGF-1 and estrogens in the intrauterine environment increase the pool of mammary stem cells *in utero*, acting as mitogens independent of BW^31,32^.

Our study had some limitations; first, the weight before and after pregnancy was self-reported, increasing the possibility of measurement error. However, data was collected in 2007 (approx. 4 years after birth), which is a shorter time interval than those reported in other studies. Second, our recruitment excluded extreme values of BW (<2500 g or >4500), which could explain the lack of evidence of an association between BW and breast composition and as a consequence the results observed in this study are only representative of girls who had normal birth weight. Third, even though we tried to control by potential confounders; such as, mother and girls weight, gyneco-obstetric data (parity, menarche), socioeconomic level (measured by maternal education), self-report gestational diabetes as a marker of maternal metabolic profile, we still might be in presence of residual confounding. For example, we did not have maternal metabolic data during pregnancy (insulin, IGF-1) and subclinical high glycemic levels during pregnancy that would be of interest to understand biological mechanisms. Also, there were unmeasured variables, like history of breast cancer, or other environmental factors that might be influencing the relationship, such as diet. On the other hand, we have longitudinal follow up that assessed the girls every six months during the pubertal time, which allowed us to determine pubertal events and progression with a better diagnosis and precision. Additionally, we used a novel approach to assess BD at young ages that is associated with a very low dose of radiation and that does not compress the breast. Finally, we evaluated BD in the daughters at the end of puberty, the time at which BD is highest and other determinants have not modified it during the life course.

Our study showed that maternal EGWG, according to 2009 IOM recommendation, increases AFGV in girls of medium-low socioeconomic level at Tanner B4 even after adjusting for potential confounding variables. However further follow up studies are needed to assess the association between EGWG and daughter’s breast cancer risk.

In Chile, we have a high prevalence of overweight and obesity during pregnancy (61%), which is an important public health concern because it affects not only the health of the mother but also that of the offspring. We were able to evaluate the effect of an EGWG in the medium term; however, it is necessary to continue the follow-up to see the future effects on the daughter’s health.

## Methods

### Study design

This study was designed as part of the Growth and Obesity Chilean Cohort study (GOCs) and the DERCAM study [“*Determinantes de Riesgo de Cáncer de Mama*” (Determinants of Breast Cancer Risk)]. Briefly, the GOCs is a Chilean cohort of 1190, who are representative of low and middle socioeconomic level children^33^. They were recruited in 2006 (mean age 3.7 y) while they were attending the National Nursery Schools Council Program in the southeastern area of Santiago, Chile. The initial aim of the cohort was to assess growth and childhood obesity and their associations with metabolic and hormonal complications in normal birth weight children^34^. The inclusion criteria were children born between 2002 and 2003, singleton birth, BW of 2.5 to 4.5 kg and absence of medical conditions that could interfere with growth (more details have been published previously)^34^. Since 2006, the children were invited to INTA periodically for the performance of different measurements and follow-up. In 2010, the girls’ mothers were invited to participate in the DERCAM study; we included only women who were premenopausal at recruitment with no history of breast cancer or other breast disease and who were not pregnant or breastfeeding (details of the study have been published elsewhere)^35^. We obtained complete data from 341 pairs of girls and their mothers of the 400 mothers who were recruited in 2010. There were no significant differences between the excluded and included girls and their mothers according to the mother’s GWG, maternal age of menarche, or the girls’ anthropometric data.

The GOCS and DERCAM study protocols and the current study were approved by the Ethics Committee Board of the Institute of Nutrition and Food Technology, University of Chile (INTA) (see related manuscript files). We confirm that all methods were carried out in accordance with relevant guidelines and regulations. Informed consents approved by the ethical committee were taken from the parents or guardian (Legally Authorized Representatives) and girls gave their assent (see related manuscript files).

### Girls’ data collection

#### Sexual maturation

Since 2009, a trained dietitian assessed pubertal development every 6 months using the Tanner scale^36^. The assessment included inspection and palpation of the breasts to differentiate between breast Tanner stage 2 (B2) and lipomastia. There was high concordance between the trained dietitian and a pediatric endocrinologist (Kappa index = 0.9)^37^.

Menarche was defined as the first bleeding or menstrual period. Menarche was assessed at all visits or by telephone through a questionnaire to differentiate menarche from other diagnoses, such as vaginal or urinary infection, or trauma, among others.

#### Anthropometry

Anthropometric data was assessed at the Tanner B4 visit by a trained dietitian using standardized protocols (ICC ≥ 0.75)^33^. Weight was measured with a portable scale, SECA model 770 (capacity of 200 kg precision of 0.1 kg); height was measured with a stadiometer, HARPENDER model 603 (capacity 200 cm, sensitivity 0.1 cm); and waist circumference, defined as the midpoint between the last rib and the iliac crest, was measured with a tape, LUFKIN W606PM (capacity 200 cm and precision 0.1 cm). We defined central obesity as a WC > the 90^th^ percentile according to age of the girls (9 to 13 y). The cutoff points for WC > the 90^th^ percentile according to NHANES III were 73.6, 76.6, 79.7, 82.7, and 85.8 cm for girls from 9 to 13 y old, respectively. Body mass index (BMI) was calculated as weight (kg)/height^2^ (m^2^), and we estimated the BMI Z-score according to the 2007 World Health Organization (WHO) data^38^.

BW was collected retrospectively from clinical records, and the quality of the BW data has been previously evaluated, indicating <1% implausible values (GOCS protocol)^39^.

#### Breast composition assessment

At the Tanner B4 visit, girls were invited to receive a breast DXA scan in order to evaluate the absolute fibroglandular volume (AFGV, cm^3^), total breast volume (BV, cm^3^) and percentage of fibroglandular volume (%FGV = AFGV/BV, %). These measurements were carried out as defined by the protocol developed by Dr. John Shepherd (more details have been published elsewhere)^40^ at INTA using a GE Lunar Prodigy Bone Densitometer (GE Healthcare). Measurements were calibrated using a N17NS phantom (a density step phantom of constant thickness consisting of seven steps of different fractions of breast fibroglandular tissue and adipose fat) to estimate AFGV and %FGV. The DXA scans were performed by a single technician trained by Dr. John Shepherd according to the standardized protocol. Briefly, girls were positioned laying on the left side of their body (lateral decubitus) with their left forearm under their head and their right hand holding their right breast to remove it from the scanning zone of their left breast. After the left breast was scanned, the girls rolled over her back to facilitate the scanning of the right breast using the same protocol. We used the mean of the right and left breast to estimate %FGV and AFGV for each girl. The total dose of radiation from the 3 scans was estimated as 45 uSv, which is approximately equivalent to 5 days of natural radiation. Raw images were saved in low and high energy, and AFGV and %FGV were calculated using software from one of the investigators (Shepherd) with intra- and interrater readings all had ICC values greater than 0.9. Recently, Pereira *et al*. found that in 200 GOCs mothers, the precision of the DXA data after the repositioning of the left breast was 2.8%, and the ICCs for %FGV and AFGV were > 0.9^19^.

### Mothers’ data collection

In 2007, the GOCs mothers were invited to INTA for an anthropometric assessment and to answer a questionnaire related to sociodemographic and gyneco-obstetric data. Mothers self-reported their pre- and post-gestational weight in kg. Height was evaluated at the time of the visit, using the same procedure as described above. Using the self-reported data, pre-gestational and post-gestational BMI was calculated. We defined GWG as the total pregnancy weight gain in kg. Additionally, based on the 2009 IOM recommendation we categorized the GWG in below, within and above the recommendation related to the pre-gestational weight BMI (WHO)^6^.

From 2011 to 2012, 402 mothers of GOCs girls were invited to receive a digital mammography [craniocaudal (CC) and mediolateral oblique (MLO) views] during the follicular phase of their menstrual cycle at a private clinic in Santiago. Raw images of both breasts were exported from the Hologic Selenia system (Marlborough, MA, USA), and AFGV and %FGV were estimated using a fully automated computer-assisted method with VOLPARA software (version 1.4.2) (Matakina Technology, Wellington, New Zealand).

### Statistical analysis

Descriptive statistics were used to characterize the predictor, confounder and outcome variables. Maternal GWG was categorized according to the pregestational nutritional status IOM 2009 guidelines: i) above GWG ii) within GWG iii) below GWG. We dichotomized GWG into two groups: i) EGWG according to pre-gestational nutritional status and ii) non EGWG (a GWG within or below the recommendations). Additionally, we used GWG in kilograms. The daughters’ breast compositions (AFGV, %FGV) were dichotomized according to their 80th percentile distribution.

Logistic regression models were used to estimate the ORs and 95% CIs comparing the three categories of GWG using breast composition at Tanner 4 and below the recommendation as the reference group. Additionally, we assessed GWG as a dichotomous variable (EGWG vs non-EGWG), GWG in kilograms, and birth weight as determinants of breast composition data at Tanner B4 (%AFGV and FGV). Models were adjusted for the daughter’s BMI Z-score, the presence or absence of menarche (yes/no) at the Tanner B4 visit (DXA measurement visit), maternal parity (1 or >1 living birth), maternal education (> or <12 y), gestational diabetes mellitus (yes/no) and %FGV measured by mammography from 2011–2012. Additionally, we adjusted for birth weight (> or <the 80^th^ percentile of the girls’ distribution) and maternal pregestational nutritional status when we assessed GWG in kg. The analysis was carried out in Stata version 13; results were considered significant if the p-value was <0.05.

## Supplementary information

Supplementary information.

## Data Availability

The datasets generated and/or analyzed during the current study are not publicly available, because the study is still ongoing, but are available from the corresponding author on reasonable request.
