# Supplementary material for: Effect of excessive gestational weight on daughters’ breast density at the end of puberty onset
Source: Sci Rep. 2020 Apr 20;10:6636. doi: 10.1038/s41598-020-63260-9 (PMC7171116; doi:10.1038/s41598-020-63260-9)
Supplement: Supplementary file 1 — Supplementary information. [file 41598_2020_63260_MOESM1_ESM.pdf]

Manuscript title: "Effect of excessive gestational weight on daughters' breast density at the end of puberty onset"

Authors: López, Ana[1]; Garmendia, María Luisa[2]; Shepherd John[3]; Michels, Karin[4,5]; Corvalán, Camila[2]; Pereira, Ana[2*].

Supplementary table S1. Association between Gestational Weight Gain (in kg and stratified by IOM) and breast composition (%FGV, AFGV): crude and adjusted models.

	**Log FGV% (ß, 95%IC)**				**Log AFGV (ß, 95%CI)**			
	**Crude model**	**Adjusted model 1**	**Adjusted model 2**	**Adjusted model 3**	**Crude model**	**Adjusted model 1**	**Adjusted model 2**	**Adjusted model 3**
**Gestational weight gain in kg. ****	-0.003 (-0.00-0.00)	0.001 (-0.00-0.00)	0.002 (-0.00-0.00)	0.002 (-0.00-0.00)	0.007 (0.00-0.01)*	0.007 (0.00-0.01)*	0.008 (0.00-0.01)*	0.007 (0.00-0.01)*
**Gestational weight gain according to IOM**								
Below	1.00	1.00	1.00	1.00	1.00	1.00	1.00	1.00
Within	-0.001 (-0.11-0.11)	0.0216 (-0.05-0.1)	0.003 (-0.09-0.1)	0.002 (-0.09-0.09)	0.03 (-0.08-0.12)	0.06 (-0.05-0.17)	0.05 (-0.1-0.2)	0.04 (-0.09-0.17)
Above	-0.08 (-0.18-0.02)	0.03 (-0.05-0.09)	0.03 (-0.06-0.11)	0.03 (-0.06-0.11)	0.08 (-0.02-0.19)	0.08 (-0.02-0.18)	0.08 (-0.04-0.20)	0.07 (-0.05-0.2)
**EGWG vs not EGWG**	-0.08 (-0.16-0.01)	0.01 (-0.05-0.08)	0.02 (-0.05-0.10)	0.03 (-0.05-0.10)	0.07 (-0.01-0.15)	0.05 (-0.03-0.14)	0.06 (-0.05-0.16)	0.05 (-0.05-0.16)

***Model was statistically significant (p value <0.05)**
Adjusted model 1 Adjusted by maternal variables: education, parity, gestational diabetes mellitus; & Girls variables at time B4: BMI Z score and presence of menarche)
Adjusted model 2: Model 1 + maternal breast density.
Adjusted model 3: Model 2 + birth weight (in kg.)

**** Gestational weight gain models were adjusted by pre gestational nutritional status.**
